# Pneumocystis jiroveci pneumonia with cytomegalovirus infection diagnosed by metagenomic next-generation sequencing in a patient with nephrotic syndrome: A case report

**DOI:** 10.1097/MD.0000000000026842

**Published:** 2021-08-06

**Authors:** Qian Yu, Xuchun Ding, Wen Wang, Yafang Lou

**Affiliations:** Respiratory Department, Hangzhou TCM Hospital Affiliated to Zhejiang Chinese Medical University, Hangzhou, China.

**Keywords:** case report, cytomegalovirus, metagenomic next-generation sequencing, Pneumocystis jiroveci pneumonia, prophylaxis

## Abstract

**Introduction:**

Opportunistic infection with multiple pathogens currently has become less uncommon since the application of immunosuppressant or corticosteroid in non- Human immunodeficiency virus patients. However, the clinical diagnosis of the co-infection remains difficult since the uncertainty and deficiency of the microbiologic testing methods.

**Patient concerns:**

A 66-year-old male patient was admitted to our hospital with chest stuffiness, shortness of breath and elevated body temperature.

**Diagnosis:**

He was diagnosed with the co-infection of Pneumocystis jiroveci and cytomegalovirus by metagenomic next-generation sequencing of bronchoalveolar lavage fluid after bronchoscopy.

**Interventions:**

The patient was empirically treated with broad-spectrum antibiotics, trimethoprim/ sulfamethoxazole and ganciclovir in the beginning of the admission.

**Outcomes:**

The condition of this patient was not improved even with the intervention at the early stage of the disease. His family requested discharge after 24 inpatient days.

**Lessons:**

This case highlights the application of metagenomic next-generation sequencing in the clinical diagnosis of pulmonary co-infection. Suitable prophylaxis, necessary clinical awareness and accurate diagnosis are indispensable for immunocompromised patients with pulmonary infection.

## 1 Introduction

Pneumocystis jiroveci (PJ) and cytomegalovirus (CMV) are both common opportunistic infection which could be detected in human immunodeficiency virus (HIV)-positive patients or the non-HIV patients with immunosuppression. Although PJ and CMV infection could be diagnosed in the early stage by the polymerase chain reaction, the clinical outcomes of the patients are still influenced by the underlying disease and its disease state, suggesting that prophylaxis and early diagnostic measures for these infections remain to be established.^[1]^ Metagenomic next-generation sequencing (mNGS), a DNA/RNA based technique with high sensibility and comprehensiveness, can greatly enhance the diagnostic accuracy of the infection especially for the patients in a serious condition. Here we describe a case of PJ and CMV co-infection in a non-HIV carrier on chronic systemic corticosteroids due to nephrotic syndrome by mNGS.

## 2 Ethical statement and consent

The patient's family member has provided informed consent for publication of the case, because the patient was already deceased after the hospital discharge. Since case reports are often considered not research and do not need institutional review board approval,^[2]^ we have not gotten ethical approval form the Ethical Committee of Hangzhou Hospital of Traditional Chinese Medicine.

## 3 Case presentation

The 66 year-old male patient was admitted to our hospital with chest stuffiness, shortness of breath and elevated body temperature. He had been receiving oral steroids (methylprednisolone 24 mg twice a day) for nephrotic syndrome, which had been diagnosed 2 months earlier. The patient had a history of arterial hypertension, hyperlipidemia, arteriosclerosis and plaque of lower limbs, carotid plaque, Schistosomiasis liver disease in 1970, resection of Anal cyst in 2020, right little finger repair due to fracture in 2000.

**Figure 1:**
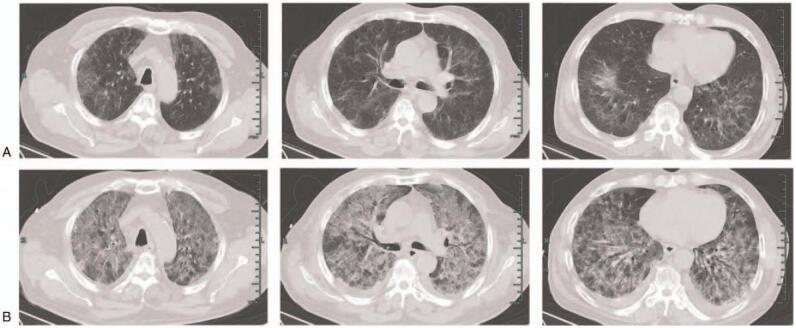
CT scan of the lung. A: On the 1st day, a CT scan showed extensive interstitial pneumonitis of 2 lung fields with the crazy-paving pattern in the middle and peripheral region of 2 upper lungs. B: On the 4th day, a CT scan showed the obvious deterioration of exudation and pleural effusion in both lungs.

On admission, the patient complained of chest stuffiness and mild dyspnea with cough and expectoration. These symptoms had started a month earlier after daily exercise every time. In 7 hours preceding admission, he had developed body temperature up to 37.8°C accompanying chills and dizziness. He denied the travel history to coronavirus epidemic areas or the contact with the coronavirus-infected people. His vital signs were stable except with 93% oxygen saturation through nasal catheter of 2 L/min. Physical examination revealed fine rales at the bottom of both lungs. No other positive signs could be found in the physical examination. The white blood cell count was 13.1 × 10^9^/L (neutrophils, 81.2%; eosinophils, 0.2%; basophils, 0.3%; lymphocytes, 13.9%; and monocytes, 4.4%). CD4+ cells count was 1.55 × 10^9^/L. The PaO_2_/FiO_2_ ratio was 213.8. The test of nucleic acid of coronavirus and IgG/IgM anti-coronavirus antibodies were both negative. High-resolution computed tomography identified extensive interstitial pneumonitis of 2 lung fields with the crazy-paving pattern in the middle and peripheral region of 2 upper lungs. (Fig. 1A). Sputum culture on admission was positive for Klebsiella pneumoniae and Candida albicans. Urine and blood cultures were negative. Serology on day 4 showed the cytomegalovirus infection with positive CMV IgG antibodies.

We started sulfamethoxazole (1200 mg) and trimethoprim (240 mg) therapy every 6 hours to treat the presumptive Pneumocystis jiroveci pneumonia (PJP) considering of the chest imaging manifestation and the application of oral steroids owing to nephrotic syndrome. We also prescribed ganciclovir 300 mg every 12 hours to treat the infection of CMV, Piperacillin/Tazobactam 4.5 g every 8 hours to cover the bacterial infection. Methylprednisolone was also administered with the dosage of 80 mg every 12 hours.

On hospital day 4, his body temperature unexpectedly elevated up to 39.5°C accompanying with the symptoms of more severe chest stuffiness and shortness of breath. Physical examination revealed increased crackles in both lungs and 90% oxygen saturation through nasal catheter of 15 L/min. He was immediately intubated and transferred to intensive care unit. High-resolution computed tomography showed the obvious deterioration of exudation and pleural effusion in both lungs (Fig. 1B). We added Caspofungin 70 mg on the first treatment day and then 50 mg every day to cover the possible fungal infection. We also prescribed human immunoglobulin 20 mg every day to enhance the immunity of the patient. Both of 1,3-beta-D-glucan test (G test) and Galactomannan test were adopted and no positive results were found. He was given a bedside bronchoscopy. PJ, cytomegalovirus and Candida albicans were isolated from bronchoalveolar lavage fluid (BALF) by mNGS with the relative abundance of 98.05%, 83.33% and 0.32% (Tables 1 and 2).

**Table 1:** mNGS report of the fungal in BALF.

Genus		Species		
Name	Sequence number	Name	Sequence number	Relative abundance
Pneumocystis	2162	*Pneumocystis jirovecii*	2162	98.05%
Candida	7	*Candida albicans*	7	0.32%

**Table 2:** mNGS report of the virus in BALF.

Name	Sequence number	Relative abundance
Human cytomegalovirus	25	83.33%

The respiratory status of this patient continued to decline despite the treatments. His family opted for palliative treatment but eventually give up the following medical care and requested discharge after 24 inpatient days. The patient died on the second day after leaving the hospital.

## 4 Discussion

This was a case of PJ and CMV co-infection. PJP is a common opportunistic and life-threatening infection leading to respiratory failure in patients with HIV or other immunocompromising reasons. Generally, there are some risk factors for the development of PJP, including immunosuppressive therapy especially corticosteroids and chemotherapy, solid organ transplantation, low CD4+ T cell counts, neutropenia and so on.^[3]^ As previously reported,^[4,5]^ PJP is more severe in HIV-negative patients comparing to HIV-positive patients, as with much higher rates of intensive care unit admission and mortality. The mortality in HIV-negative patients can reach up to 40%, which is twice as high as in HIV-positive patients with PJP and this may due to their enhanced inflammatory response as a result of the more intact immune system.^[6]^ Thus, the prophylaxis of PJP is particularly significant. It is recommended that PJP prophylaxis should be administered to the patients receiving the equivalent of at least 20 mg prednisone daily for 4 weeks or more.^[7]^ In this case, corticosteroid usage suppressed the immune system of the patient, therefore rendering him susceptible to PJP. However, negative results of G test and Galactomannan test of this patient didn’t offer us any evidence of fungal infection. To the best of our knowledge, the incomplete international guidelines or consensus for prophylaxis of PJP in non-HIV patients made it challenging in identification of the proper time to start prophylaxis at an earlier stage of risk for these vulnerable patients.^[8]^ What's worse, the conventional tests sometimes are neither non-specific nor unavailable in some countries.^[9]^ These all lead to the difficulties in diagnosis and treatment of PJP.

CMV is a kind of prevalent human herpesvirus with non-dominant clinical syndrome in immunocompetent people, more likely being concomitant with the occurrence of PJP.^[10,11]^ In immunocompromised patients, either the reactivation of latent CMV acquired in early life or primary infection in previously uninfected individuals could result in CMV infection.^[12]^ Patients experiencing CMV viremia are at an increased risk of subsequent development of invasive fungal disease like PJP because of the common shared clinical risk factors including immunosuppression, corticosteroids, graft-versus-host disease and graft rejection.^[13]^ The patient in this case turned out to be the co-infection of PJ and CMV. It is controversial about the final results and medicine intervention of CMV and PJ co-infection. Tark et al found that the morbidity and mortality of PJP were not significantly different in the patients with and without pulmonary CMV infection thus it is unnecessary to prescribe an anti-CMV regimen when CMV is co-isolated from the BALF in patients with PJP.^[14]^ However, Pervin et al revealed the increased risk of mortality in the setting of CMV and PJ co-infection in non-HIV but immunocompromised patients.^[15]^ Lai CC et al also found that CMV and PJ co-infection is the risk factor for 30-day mortality in those patients with medication of corticosteroid and immunosuppressant.^[16]^ More evidence proclaimed the indispensability of antiviral treatment based on the poor prognosis of CMV infection.^[17]^ The varied results may depend on the difference of sample numbers, testing methods and specimen collection in these studies. We administered ganciclovir to treat CMV infection at the very early stage of the course of disease. Unfortunately, the combined treatment of sulfamethoxazole/trimethoprim and ganciclovir didn’t stop the progression of the disease. To this respect, large-scale evidence-based medicine studies are still necessary to discuss the effectiveness of the co-treatment.

mNGS is an unbiased and non-invasive approach that allows for direct and universal pathogen detection including viruses, bacteria, fungi and parasites from clinical specimens.^[18]^ Comparing with the conventional molecular test, mNGS has the following advantages:^[19]^ it is a culture-independent approach for identification for all pathogens; a single protocol is sufficient in detecting the pathogens; there is no need for presumed or target primers; it could be used to study antibiotic resistance genes. Depending on the constantly refined sequencing instruments, relatively reduced sequencing costs and comprehensive genomic database, mNGS is considered to be a key driver for precision diagnosis of infectious diseases, especially in the most difficult-to-diagnose cases or for immunocompromised patients.^[20]^ Of note, mNGS technology has already been used for detecting infection of the immunosuppressed patients with dermatomyositis, febrile neutropenia or pneumonia under chemotherapy or transplant.^[21,22]^ With high sensitivity and efficiency, mNGS could be regarded as an effective and promising technique for accurate diagnosis of mixed pulmonary infection especially in those negative cases identified by conventional test.^[23]^ In this case, G test showed the negative result. While the BALF sample of the patient was analyzed through mNGS within 72 hours with the result of co-infection of PJ, CMV and Candida albicans. But Candida albicans is not regarded as the pathogen being responsible for respiratory failure of this patient because of the low abundance tested by mNGS.

To date, there have been only 2 retrospective studies on successful use of mNGS in diagnosing mixed pulmonary infection involving PJ and CMV in non-HIV patients (Table 3).^[23,24]^ These studies give us broader point of view in diagnosis approach. Our report is the first clinical case demonstrating the explicit role of mNGS in diagnosis of co-infection of PJ and CMV in HIV-negative patient. This case highlights not only the necessity of prophylaxis of opportunistic infection in immunocompromised patients, but also the function of mNGS in identification of multiple pathogens. In this case, the empirical and standardized anti-PJ and anti-CMV treatments may lead to limited value of mNGS to some extent, however, this technique is nonetheless of great assistance and can better guide the appropriate therapy to the immunocompromised patients in whom the spectrum of potential pathogens is greater. Regrettably is the discontinuation of the treatments that made it impossible for us to observe the patient's condition and eventually hastened his death.

**Table 3:** Clinical studies of co-infection of PJ and CMV tested by mNGS in non-HIV patients.

Author/Year	Case number of co-infection of PJ and CMV	specimen	Results of mNGS	Underlying disease
Ying L/2020	2	BALF	Nocardia sp. CMV PJ	chronic pulmonary diseases and immunosuppression
Jiahui W/2019	5	BALF	CMV PJ	Hematological system diseases

## 5 Conclusion

In summary, we have described this case to emphasize the importance to raise clinical awareness of co-infection of PJ and CMV in immunosuppressed patients. Suitable prophylaxis should be administered and sometimes indispensable during the routine treatment. Moreover, mNGS is a key driver for precision diagnosis of infectious diseases with the features of individualization and comprehensiveness. Hopefully, the improvement of accuracy would enable mNGS to be more convenient and reliable in clinical work.

## Acknowledgments

The case report is not funded by any foundation and the authors declare that they have no competing interests.

## Author contributions

**Conceptualization:** Qian Yu.

**Data curation:** Xuchun Ding.

**Methodology:** Wen Wang.

**Writing – original draft:** Qian Yu.

**Writing – review & editing:** Qian Yu, Yafang Lou.
